# The Role of Tenascin-C in the Physiopathology of Familial Mediterranean Fever

**DOI:** 10.7759/cureus.64067

**Published:** 2024-07-08

**Authors:** Emin Guluzade, Berna Güzel, Demet Yalcin Kehribar, Muhammed Okuyucu, Metin Özgen, Bahattin Avcı

**Affiliations:** 1 Internal Medicine, Medicana Hospitals, İstanbul, TUR; 2 Internal Medicine, Samsun Alaçam State Hospital, Samsun, TUR; 3 Internal Medicine, Dokuz Eylul University Faculty of Medicine, İzmir, TUR; 4 Internal Medicine, Ondokuz Mayıs University Faculty of Medicine, Samsun, TUR; 5 Rheumatology, Ondokuz Mayıs University Faculty of Medicine, Samsun, TUR; 6 Biochemistry, Ondokuz Mayıs University Faculty of Medicine, Samsun, TUR

**Keywords:** rheumatology, physiopathology, tenascin-c, inflammation, familial mediterranean fever

## Abstract

Objective: Familial Mediterranean fever (FMF) is an autoinflammatory disease common in the Mediterranean basin. It has been determined that tenascin-C level is increased in rheumatic inflammatory diseases such as rheumatoid arthritis (RA), systemic lupus erythematosus, and systemic sclerosis. However, the role of tenascin-C has not been investigated in FMF. This study aimed to investigate serum tenascin-C levels in FMF patients and to investigate possible relationships between them.

Materials and methods: About 38 patients diagnosed with FMF and 40 healthy controls were included in the study. The patient’s sex, age, clinical symptoms, physical examination, and laboratory results were recorded. Serum tenascin-C levels were determined by the enzyme-linked immunosorbent assay (ELISA) method.

Results: The serum tenascin-C levels were significantly lower in the FMF patients (10297 ± 8107 pg/ml) compared to the healthy control group (29461 ± 13252 pg/ml) (p < 0.001). In receiver operating characteristic (ROC) analysis, when the cut-off point was chosen as 11076 pg/ml, sensitivity was 77.1% and specificity was 91.9%. When the cut-off point was chosen as 19974 pg/ml, sensitivity was 91.4% and specificity was 75.7%. It was determined that the serum tenascin-C levels did not correlate with age, gender, and laboratory parameters in the healthy control group and FMF patients (p > 0.05).

Conclusion: This is the first study investigating tenascin-C levels in FMF. Tenascin-C levels in FMF patients were lower than in healthy controls. Low tenascin-C levels in FMF, which are high in other chronic rheumatic diseases, may be a valuable indicator. Therefore, serum tenascin-C level seems to be a useful marker in distinguishing FMF patients from healthy individuals.

## Introduction

Familial Mediterranean fever (FMF) is characterized by recurrent and self-limiting episodes of fever and these fever episodes are accompanied by at least one of the following: aseptic peritonitis, arthritis, pleural fluid, and erysipelas-like erythema [1]. FMF is the most prevalent autoinflammatory disease with autosomal recessive inheritance [2,3]. The MEFV gene, which has been considered associated with FMF, was identified by positional cloning in 1997. MEFV gene is located on chromosome 16p13.3 and consists of 10 exons and 781 codons [4,5]. Five MEFV gene mutations (M680I, M694V, M694I, V726A, and E148Q) account for 85% of FMF-related conditions. The protein called pyrin/marenostrin, a gene product, is expressed from polymorphonuclear cells and monocytes and regulates the inflammatory response [5].

Tenascin-C is an extracellular matrix protein that is minimally expressed in healthy tissues but shows increased expression in response to tissue damage. Typically, its expression is transient with protein being cleared until the completion of tissue repair. Tenascin-C expression level is decreased in normal tissues, while it is increased in cases of wound healing with remodeling and neo-vascularization, inflammation, and malignancies [6]. Animal experiments suggested that tenascin-C had an important role in the development of fibrosis [6,7]. However, persistent expression of tenascin-C is linked to various pathological conditions, including rheumatoid arthritis (RA), scleroderma, and some fibrotic diseases [8-11].

Despite reports of elevated tenascin-C levels in rheumatologic diseases like RA, ankylosing spondylitis (AS), and scleroderma, as well as in other chronic diseases, no prior research has examined its role in autoinflammatory diseases [12-14]. The present study aimed to investigate the role of tenascin-C in FMF, the most prevalent autoinflammatory disease. It may seem that tenascin-C might also be involved in the pathogenesis of FMF.

## Materials and methods

Participants

This study included 38 (26 women, 12 men) patients aged between 18 and 65 years, without chronic diseases, who presented to our Rheumatology Outpatient Clinic at Ondokuz Mayıs University Faculty of Medicine, Samsun, Turkey, between January 1, 2021, and January 1, 2022, and were diagnosed with FMF based on Tel Hashomer criteria. Patients with pregnancy, ongoing breastfeeding, active infection recent surgeries, other autoimmune conditions, or use of medications that might affect inflammatory markers were excluded from the study. The control group included 40 healthy volunteers aged above 18 years, without any chronic disease, active infection, or pregnancy. Ethical approval was obtained from Ondokuz Mayıs University Clinical Research Ethics Committee (dated August 22, 2022, number B.30.2.ODM.0.20.08/765-908-594 and decision number OMU KAEK 2021/492). The study was conducted in accordance with the principles of the Declaration of Helsinki. All participants provided written informed consent prior to participation.

Procedures

Patients’ sex, age, age at diagnosis, family history, clinical symptoms (fever, peritonitis, pericarditis, pleuritis, erysipelas-like erythema, myalgia, arthritis), physical examination, laboratory results (tenascin-C, erythrocyte sedimentation rate (ESR), C-reactive protein (CRP), white blood cell (WBC), hemoglobin, platelet, urea, creatinine, aspartate aminotransferase, and alanine aminotransferase values) and previous treatments were recorded.

Laboratory analysis

Peripheral venous blood samples from both groups were centrifuged at 3000 RPM for 15 minutes and sera were stored at -80˚C. The samples were thawed at room temperature and studied on the day of examination.

The levels of tenascin-C in serum samples from both the patient and control groups, stored at -80˚C, were assessed utilizing a suitable commercial ELISA kit (HUMAN Tenascin-C ELISA kit Sunred Biological Technology Co. No. 201-12-1415, Shanghai, China). Enzymatic reactions were measured in an automated microplate photometer. Tenascin-C concentrations were determined upon the comparison of the optical density of the samples to the standard curve. The measurement range of the kit was 200-60000 pg/mL. All the measurements were performed pursuant to the instructions of the respective manufacturers.

Statistical analysis

IBM SPSS Statistics for Windows, Version 22 (Released 2013; IBM Corp., Armonk, New York, United States) program was used for the purposes of statistical analysis. Numeric variables were presented as the average value along with the standard deviation (SD). Demographic data and clinical findings of the patients enrolled in the study were analyzed to determine both numeric values and percentages. The Student’s t-test was used in variables with normal distribution, with an aim to investigate the association between clinical findings in FMF and tenascin-C level. Pearson's correlation test was used for variables with normal distribution, and Spearman's correlation test was used for variables without normal distribution to investigate the relationship between the data. Receiver operating characteristic (ROC) analysis was conducted to evaluate the diagnostic significance of tenascin-C. A p-value below 0.05 was deemed statistically significant. Unless otherwise stated, values are presented as mean ± SD.

## Results

**Figure 1:**
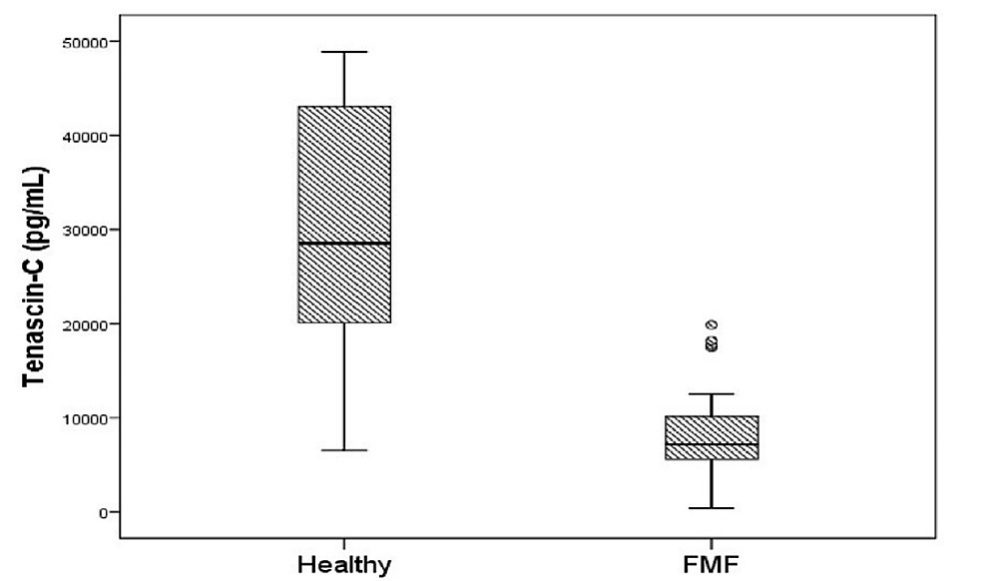
Tenascin-C levels in familial Mediterranean fever (FMF) and control groups

A total of 78 people, including 38 patients in the FMF group and 40 people in the healthy control group, were included in the study. Upon comparison of the laboratory values from the patient and control groups, there was a statistically significant difference between the two groups by ESR value (p < 0.001). There were no statistically significant variations between groups in terms of age, gender, CRP, hemoglobin, platelet count, creatinine, aspartate aminotransferase, and alanine aminotransferase levels. The demographic information and laboratory results from the FMF and healthy control groups included in the study are shown in Table 1. Tenascin-C levels were notably reduced in the FMF group (10297 ± 8107 pg/mL) in comparison to the healthy control group (29461 ± 13252 pg/mL) (p < 0.001, Figure 1).

**Table 1:** Demographic information and laboratory results from the FMF group and control group *p < 0.05 **p < 0.001 SD: standard deviation; FMF: familial Mediterranean fever; ESR: erythrocyte sedimentation rate; CRP: C-reactive protein; AST: aspartate aminotransferase; ALT: alanine aminotransferase

	Healthy controls (n = 40) Mean ± SD	FMF (n = 38) mean ± SD	t	p
Age (year)	29.2 ± 5.3	33.4 ± 14	-1.834	0.08
Sex (n) (F/M)	24/16	26/12	-1.117	0.28
ESR (mm/h)	13.2 ± 8.1	27.1 ± 20	-6.925	<0.001^**^
CRP (mg/L)	4.9 ± 5.3	13.5 ± 27.9	-1.842	0.06
AST (U/L)	22.6 ± 15.7	24 ± 12.6	-0.402	0.68
ALT (U/L)	19.1 ± 13.3	26.1 ± 19	-1.840	0.06
Hemoglobin (gr/dL)	13.9 ± 1.7	13.4 ± 1.6	-1.438	0.13
Creatinine (mg/dL)	0.79 ± 0.1	0.72 ± 0.2	-1.436	0.1
Leukocyte (/mm^3^)	6725.8 ± 1576	7119.7 ± 2448	-1.436	0.4
Platelet (x10^3^/mm^3^)	263.3 ± 46.8	275.8 ± 72.6	-0.982	0.37
Tenascin-C (pg/mL)	29461 ± 13252	10297 ± 8107	-6.924	<0.001^**^

No significant difference was found in correlation analysis between tenascin-C levels and demographic data or laboratory findings in the study groups (p > 0.05, for all) (Table 2).

**Table 2:** Correlation analysis between tenascin-C levels and demographic information and laboratory results from the study groups ESR: erythrocyte sedimentation rate; CRP: C-reactive protein; AST: aspartate aminotransferase; ALT: alanine aminotransferase

Parameter	R-value	p
Age	-0.126	0.273
ESR	-0.104	0.367
CRP	-0.197	0.084
AST	0.072	0.547
ALT	-0.079	0.492
Creatinine	0.051	0.659
Hemoglobin	-0.021	0.853
Leucocytes	0.057	0.621
Platelet	0.047	0.686

In the FMF group, there was no association observed between tenascin-C levels and age. Chi-squared tests suggested that there was no significant correlation between sex, clinical symptoms, previous treatments, and tenascin-C levels (p > 0.05, for all). In the FMF group, four patients were in the attack period. There was no statistically significant difference between tenascin-C levels in FMF patients who presented during the attack period and patients who did not present during the attack period (p = 0.203).

**Figure 2:**
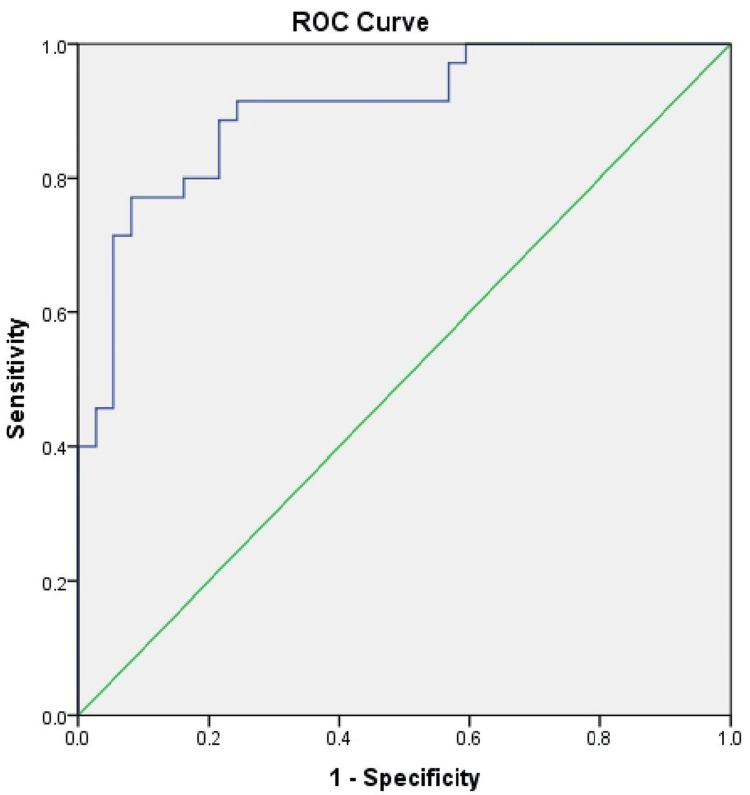
ROC curve of tenascin-C ROC: receiver operating characteristic

In ROC analysis, sensitivity was 77.1% and specificity was 91.9% with a cut-off point selected as 11076 pg/mL. For a cut-off point of 19974 pg/mL, sensitivity and specificity were 91.4% and 75.7%, respectively (for both AUC: 0.900, p < 0.001) (Figure 2).

## Discussion

This study investigated the relationship between tenascin-C level and the pathophysiology of FMF disease. Consequently, the level of tenascin-C was found to be lower in FMF patients compared to the healthy controls. Additionally, there was no correlation between tenascin-C level and disease activity or inflammatory markers. Previous studies in the relevant literature investigated autoimmune diseases and tenascin-C levels, yet this is the first study on the relationship between FMF and tenascin-C.

Bubova et al. noted elevated serum tenascin-C levels in individuals with AS relative to healthy controls. While they proposed that tenascin-C levels could indicate chronic structural spinal alterations, they did not find a correlation between tenascin-C levels and disease activity [7]. Gupta et al. examined the levels of tenascin-C in both serum and synovial fluid of individuals with AS and found that serum levels of tenascin-C were elevated in AS patients compared to those in healthy controls. As a result, it was concluded that serum tenascin-C levels were elevated in AS and could be used as an inflammation marker during the early stages of the disease [12]. In contrast, serum tenascin-C level was not increased in FMF, an inflammatory disease, in this study.

Chevalier et al. studied the expression of tenascin-C in the articular cartilage of individuals with RA and osteoarthritis. They observed higher levels of tenascin-C in the synovium affected by arthritis compared to normal synovium [15]. Another study by Chevalier et al. suggested that the occurrence and distribution of tenascin-C in normal and osteoarthritic cartilage was affected by IL-1 beta [16]. Cutolo et al. stated that there was a correlation between tenascin-C levels and joint erosions in individuals diagnosed with RA [17]. In the present study, there was no increase in tenascin-C levels in patients with FMF. While arthritis is also seen in FMF, its prevalence is much lesser. Furthermore, sequelae other than hip arthritis are not expected in FMF. The level of tenascin-C may also account for this difference between the nature of the two diseases.

A study conducted by Závada et al. found that there was no notable distinction in serum tenascin-C levels between patients diagnosed with systemic lupus erythematosus (SLE) and healthy individuals. However, as the severity of the disease escalated, there was a significant rise in tenascin-C levels. As a result, it was suggested that tenascin-C was not disease-specific, but could have indicated SLE activity and predicted the need to increase immunosuppressive treatment [18]. However, in the current study, the levels of tenascin-C were notably diminished in FMF patients compared to the healthy control group.

El-Karef et al. found that mice lacking tenascin-C exhibited reduced fibrotic and inflammatory responses compared to wild-type mice in an immune-mediated hepatitis model induced by intravenous injections of concanavalin A [19]. Moreover, administration of recombinant tenascin-C resulted in enhanced migration of hepatic stellate cells and stimulated the production of type I collagen via signaling pathways involving TGF-β1 and α9β1 integrin [20]. Ultimately, tenascin-C triggers inflammatory and fibrotic reactions and induces the progression of liver fibrosis by increasing the aggregation of hepatic stellate cells and the release of cytokines and TGF-β during the progression of hepatic fibrosis [19]. Brissett et al. suggested that tenascin-C level was high in affected tissues in patients with scleroderma, which was associated with increased IL-4 levels [21]. Another study investigated the pathogenic role of endogenous toll-like receptor (TLR) activators and tenascin-C association in fibroblasts of patients with scleroderma and mouse models of organ fibrosis. There were elevated expression levels of tenascin-C in scleroderma fibroblasts and fibrotic skin tissues from mice. According to reports, the introduction of external tenascin-C prompted the expression of collagen genes and the differentiation of myofibroblasts via TLR4 signaling. Additionally, studies demonstrated that the weakening of skin and lung fibrosis occurred, and the resolution of fibrosis was accelerated in mice lacking tenascin-C [22].

Previous studies reported that tenascin-C was involved in wound healing and tissue fibrosis [22]. End-stage organ damage develops in the involved organs and fibrotic tissue replaces normal tissue in SLE and scleroderma. Similarly, synovial fibroblasts induce permanent joint damage in RA. In contrast to AS, RA, SLE, and scleroderma, FMF does not have chronic progress, yet is characterized by attacks and the patient would not have any complaints during the inter-attack period and inflammatory markers would be within the normal range. Fibroblasts do not play an important role and end-stage organ damage does not occur in the internal organs in the pathogenesis of FMF, and kidney and lung fibrosis have not been suggested in association thereto. The fact that tenascin-C level is not increased in patients with FMF in contrast to other chronic inflammatory rheumatic diseases may indicate that it does not have a chronic fibrotic nature.

A previous study on Behçet's disease did not report an increase in tenascin-C levels [23]. In contrast with other chronic autoimmune diseases, Behçet's disease is characterized by exacerbations and there is no previous study in the relevant literature, which suggested that the fibrotic process was at the forefront of disease pathogenesis. As regards the results of the present study, tenascin-C level was even lower in patients with FMF compared to healthy controls. FMF and Behçet's disease are similar in that they do not have a chronic course. There is no chronic inflammation in these two diseases. In other words, the characteristic feature of these two diseases is that they progress with attacks. It is interesting that although tenascin-C levels increase in chronic inflammatory diseases, its levels decrease in diseases with exacerbation. Based on these results, it may be speculated that tenascin-C protein is associated with the chronicity of inflammatory conditions. An increase or decrease in tenascin-C may contribute to the chronicity of diseases.

Our study has certain limitations. This study was designed as a cross-sectional study and therefore it is insufficient to establish a cause-and-effect relationship. Certain factors, including obesity and drug use, which are thought to affect tenascin-C levels, could not have been isolated from the study. The number of patients in our study was not sufficient for subgroup analyses. The data from our study would have been stronger if a chronic autoimmune disease group was also created for the patient control group.

## Conclusions

In conclusion, previous studies reported an increase in serum tenascin-C levels in chronic autoimmune diseases, including RA, SLE, and scleroderma, yet tenascin-C levels do not increase in the autoinflammatory FMF disease. However, being the first study to evaluate tenascin-C levels in FMF patients and noting that while these levels are high in other chronic rheumatic diseases, they are low in FMF, which is an autoinflammatory condition, makes the study significant. Consequently, tenascin-C level may be a helpful marker in diagnosing FMF disease.
